# Effect of Fe Content on Microstructure and Properties of Laser Cladding Inconel 625 Alloy

**DOI:** 10.3390/ma15228200

**Published:** 2022-11-18

**Authors:** Weidong Liu, Lei Li, Guofa Mi, Jincai Wang, Yujia Pan

**Affiliations:** 1 School of Materials, Shanghai Dianji University, Shanghai 201306, China; 2 School of Materials Science and Engineering, Henan Polytechnic University, Jiaozuo 454000, China; 3 Shanghai Jinbo Laser Technology Co., Ltd., Shanghai 201306, China

**Keywords:** laser cladding, grain size, crystal orientation, high temperature wear resistance, high temperature corrosion resistance

## Abstract

Dilution rate is one of most important factors influencing the microstructure and performance of the laser cladding layer. In order to obtain a reasonable dilution rate in the laser cladding layer of Inconel 625 alloy, the laser cladding layers with different Fe content were prepared on the surface of 20# steel by the laser cladding technique. The influence of Fe content on the microstructure and performance of Inconel 625 alloy cladding layer was investigated. The results indicate that with the increase in Fe content in the alloy, the grain size of the cladding layer becomes coarser, the grain orientation difference increases first and then decreases, and the grain boundary angle decreases first and then increases. The hardness, high temperature wear resistance, and high temperature corrosion resistance gradually decreased. It is concluded that the dilution rate of Fe in laser cladding Inconel 625 alloy should be under 5 wt.%.

## 1. Introduction

Corrosion, wear, fatigue, and other failure forms are serious problems that metal materials are confronted with all along. Frequently, repairing, replacing, or even shutting down the relevant equipment due to the failure of the metal components severely limits the productivity of the manufacturing industry and raises the cost of manufacturing [1]. Modern surface engineering techniques can provide superior functional surfaces or coatings to achieve the purpose of surface modification, thereby improving the performance of remarkably high hardness, wearability, and corrosion resistance, thereby extending the service life [2,3]. In terms of strengthening and repairing of engineered surfaces, laser cladding technology can satisfy the specific performance requirements of the material surface, which is not restricted by the size or shape of parts, but also has high controllability [4]. As a newly developed additive remanufacturing technique, it has been widely applied to the surface modification of metal materials [5,6].

Compared with other surface modification techniques (such as vapor deposition, resurfacing welding, thermal spraying, electroplating, etc.), laser cladding has the advantages of low dilution rate, thin cladding layer in the heat-affected zone of the matrix, controllable thickness, environmental protection, etc. [7,8,9,10]. The laser beam has good coherence and high energy density, which makes it possible to rapidly heat and cool the laser molten pool. Owing to fast solidification, the effect of solute retention is differs greatly from equilibrium solidification, and the degree of segregation can be avoided or greatly reduced, which can greatly improve the solid-solution limit of the alloy [11]. Moreover, when the molten pool is cooled, there is a large degree of undercooling, so that nanoparticles with dispersion enhancement can be obtained, and even the amorphous structure [12], which makes the cladding layer have excellent wear resistance and high hardness. Al-sayed [13] laser-claded NiCrBSiC-60wt.%WC composite layers; it was found that with the increasing of powder feeding rate, the dilution between the cladding layer and matrix was weakened. Machine learning simulation results were consistent with the experimental results. Singh [14] studied NiCrBSiC-50wt.%WC composite layer of laser cladding. The results showed that a high scanning speed, high power of the laser beam, and a moderate powder feeding rate were beneficial to improve the wear resistance of the NiCrBSiC-50wt.%WC laser cladding layer. Duriagina [15] investigated the effect of process parameters on the microstructure and performance of ferrite AISI 420 and austenite AISI 304 laser cladding layers, and obtained the process parameters with the best wear resistance and analyzed the wear resistance mechanism. Sun [16] studied the influence of adding WC-12Co on the microstructure and wear resistance of laser cladding Inconel 625 alloy, and it was found that adding WC-12Co could significantly improve the hardness and wear resistance of the Inconel 625 alloy coating. Ma [17] investigated the grain-boundary characteristic distribution of Inconel 625 alloy, and it was proved that optimum distribution of grain boundaries could improve the intergranular corrosion resistance of Inconel 625 alloy and the surface performance of nodular cast iron. Tong [18] found that by laser cladding Inconel on nodular cast iron 625 alloy coating, cladding layer, and matrix in metallurgical bonding, the bonding strength, microhardness, and corrosion resistance were significantly improved compared with the matrix. Wang [19] studied the influence of steady-state magnetic field on the Fe distribution in Inconel 718 laser cladding layer by simulation and experiment. Both the simulation and the experimental results indicate that a steady-state magnetic field can inhibit the flow of molten pool, influence the heat and mass transfer of laser cladding pool, and change the distribution of Fe elements in the cladding layer. Xue [20] carried out process research on laser cladding of Inconel 625 alloy and found that the cladding layer without stomatal cracks and other defects can be prepared within a certain process window, which indicates that the cladding technology of Inconel 625 alloy is excellent.

To obtain a good metallurgical bonding effect between the coating and the matrix, a suitable amount of surface matrix material should be used to dilute the cladding layer during the laser cladding process. However, the dilution rate has a direct influence on the microstructure and performance of the cladding layer [3].

If the dilution rate is too low, the bonding strength between the coating and the matrix will be too weak. If the dilution rate is too high, the composition of cladding material will change, obviously, and the original performance of alloy powder will be weakened, and the tendency of cracks in cladding layer will be increased, even spalling [21,22]. Xu [23] and Du [24] predicted the influence of dilution rate on the microstructure and performance of the cladding layer through machine learning and numerical simulation methods, and it was found that a reasonable dilution rate is crucial to the control of microstructure and performance of the cladding layer. Liu [25] investigated the dilution rate of laser cladding ultrahigh strength steel. It was found that the dilution rate had a certain effect on the softening of the heat-affected zone, and the dilution effect of the substrate on the cladding layer improved the hardness of the fusion zone, but the higher the dilution rate, the lower the crack resistance of the fusion zone.

Inconel 625 alloy has a melting point of 1290~1350 °C, has excellent corrosion resistance to inorganic acid, and has a wide range of applications. However, nickel-base alloys are more expensive in price. Therefore, a layer of Inconel 625 alloy is laser cladded onto the surface of the base material to improve the surface modification and greatly reduce the cost. In this study, Inconel 625 alloy cladding layer was prepared by laser cladding technique onto the surface of 20# steel, and the influence of Fe content on the microstructure and performance of the Inconel 625 alloy cladding layer was investigated. Results provide guidance for the control of the Fe dilution rate of Inconel 625 alloy by laser cladding technology.

## 2. Experimental Procedure

**Figure 1:**
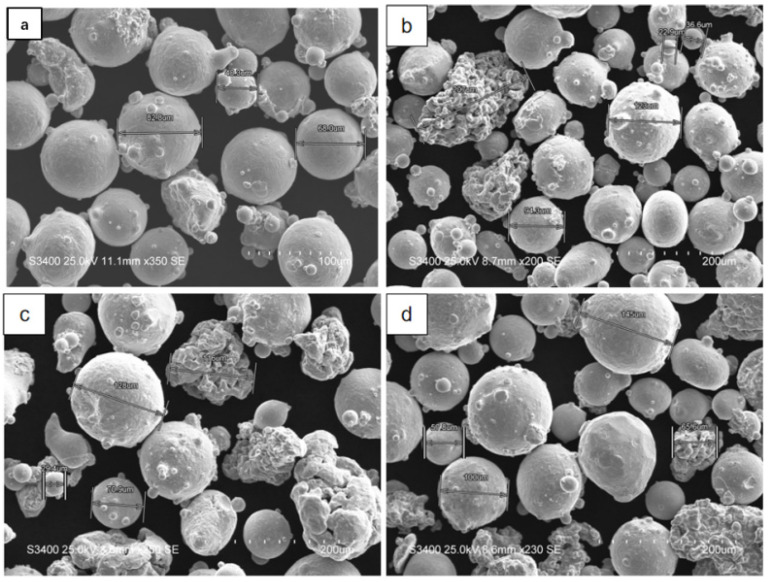
Morphology and size of laser cladding powder materials: (**a**) Inconel 625; (**b**) 5 wt.% Fe; (**c**) 10 wt.% Fe; (**d**) 15 wt.% Fe.

Four types of cladding powder materials were prepared: Incone l625; Inconel 625 + 5 wt.%Fe; Inconel 625 + 10 wt.%Fe; and Inconel 625 + 15 wt.% Fe. (Inconel 625, 5 wt.% Fe, 10 wt.% Fe, 15 wt.% Fe represent the following four cladding layers). The cladding powder was prepared by the vacuum atomization method, and its chemical composition is shown in Table 1. The average diameter of spherical Inconel 625 alloy powder is 110 μm and the mean diameter of the irregular shape Fe powder is 120 μm; the morphology under scanning electron microscope is shown in Figure 1. The base material is 20# steel, the chemical composition of which is shown in Table 2. Before the experiment, the powder was placed in a 120 °C vacuum oven for 2 h to ensure the dryness of the powder and to avoid defects such as stoma oxide caused by moisture in the cladding layer. The 100 mm × 100 mm × 10 mm base material was polished with sandpaper to remove the oxide layer, and then washed with acetone in an ultrasonic cleaning machine to remove oil and impurities.

**Figure 2:**
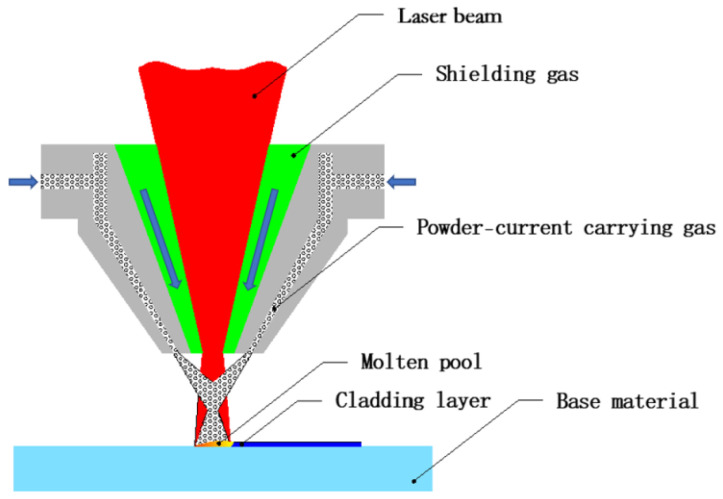
Schematic diagram of coaxial powder feeding.

The heat source was a German Laserline LDM6000-100 laser, the laser wavelength was 1070 μm, the core diameter of the fiber was 600 μm, and the laser-cladding-head focal length was 267 mm. The laser power was 2000 W, the feeding rate of the powder was 30 g/min, the laser scanning speed was 10 mm/s, and the lapping rate was 35%. High purity Ar (≥99.9%) was used as the powder carrier gas and guard gas. The carrier gas flow rate was 9 L/min and the guard gas flow rate was 15 L/min. As illustrated in Figure 2, the laser cladding was performed by coaxial powder feeding. The principle is as follows: metal alloy powder is converging on the surface of the substrate at a uniform velocity through the coaxial powder feeding nozzle under the action of current-carrying gas, and the size of the powder is identical with the size of the laser spot. Moreover, the high-energy laser beam is focused on the center of the powder spot, so that the powder and matrix can melt rapidly to form a laser molten pool. When the spot leaves the molten pool by scanning forward at a uniform velocity, the molten pool is quenched and solidified, and the cladding material and a very small amount of matrix material form a cladding layer combined by metallurgy. Using a YASKAWA MOTOMAN-SP100 six-axis industrial robot, the laser scanning movement and control are implemented. In order to isolate the influence of thin-action release of Fe element in the matrix for the experiment, the multilayer cladding method was adopted in this study.

The sample was cut by a wire cutting machine with an electric spark and prepared according to the requirements, and then tested with backscattered electron diffraction (EBSD), X-ray diffraction (XRD), microhardness, high-temperature wear resistance, and high-temperature corrosion resistance. The influence of Fe content on the microstructure and performance of the laser cladding layer of Inconel 625 alloy was investigated.

## 3. Results and Discussion

### 3.1. Phase Analysis

**Figure 3:**
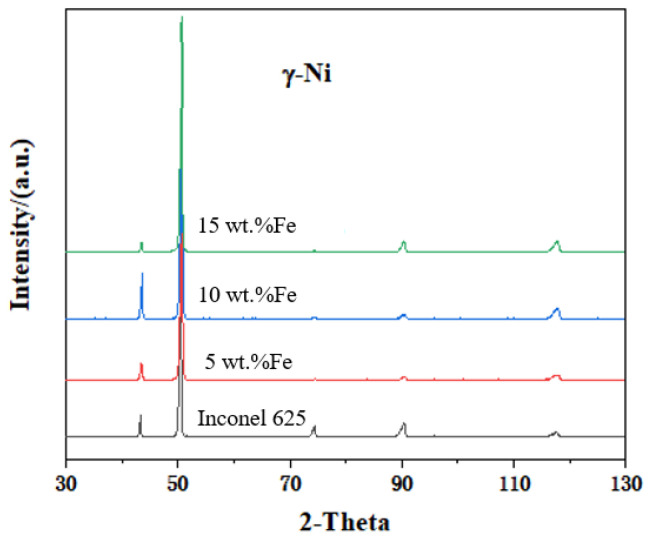
XRD patterns of Inconel 625 alloy cladding with different Fe contents.

The crystal structures of the cladding layers were analyzed by a D8 X-ray diffractometer (Cu target Kα ray scanning, scanning range 30°~130°, scanning rate 5°/min). The XRD patterns of the cladding layers are shown in Figure 3.

All of the cladding layers are γ-Ni; the increase in Fe content does not result in the formation of a new phases. The crystal structure of Inconel 625 alloy is less sensitive to Fe content, and it is more stable in phase.

### 3.2. EBSD Analysis

**Figure 4:**
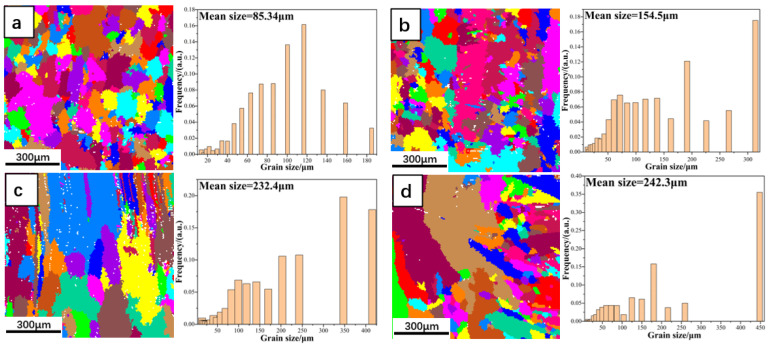
Grain size distribution of Inconel 625 alloy cladding with different Fe contents: (**a**) Inconel 625; (**b**) 5 wt.% Fe; (**c**) 10 wt.% Fe; (**d**) 15 wt.% Fe.

When the composition of the material is changed, the thermodynamics and kinetics of solidification change accordingly, and the dendrite growth is disturbed. The grain size and distribution of the cladding layer with different Fe content are analyzed statistically by SEM in a backscattering electron diffractometer, and the results are shown in Figure 4.

It is found that with the increase in Fe content, the average grain size of the cladding layer is 85.34, 154.5, 232.4, and 242.3 μm, respectively. The grain size difference of the same cladding layer increases gradually; that is, the uniformity of grain size distribution is reduced. The statistical distribution histogram of grain size indicates that the increase in average grain size is mainly due to the coarsening of high proportion grains.

**Figure 5:**
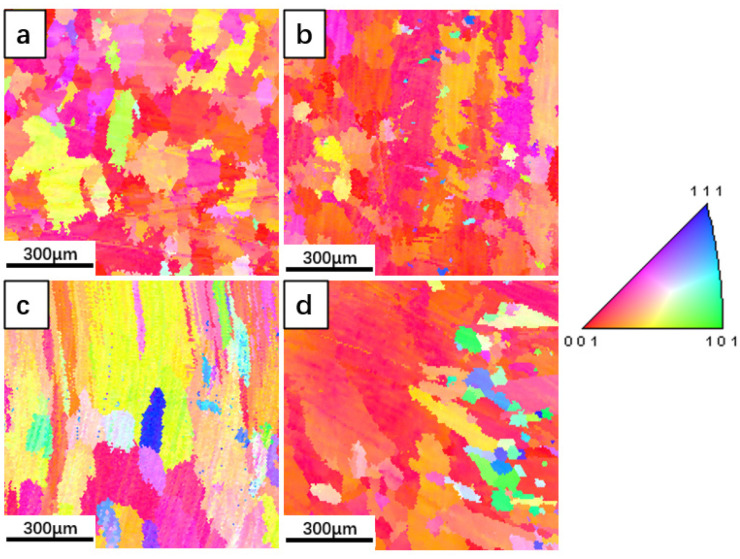
Grain orientation of Inconel 625 alloy cladding with different Fe content: (**a**) Inconel 625; (**b**) 5 wt.% Fe; (**c**) 10 wt.% Fe; (**d**) 15 wt.% Fe.

Owing to the anisotropy of interfacial tension, crystal orientation is preferred during growth; <001> is the fastest growth direction [26]. Figure 5 shows the grain orientation distribution for each cladding layer. The grain orientation of Inconel 625 alloy cladding layer is almost <001>. However, with the increase in Fe content, the grain orientation of the cladding layer increases first, and then decreases, and the orientation difference of 10 wt.% Fe grains is the largest. Grain coarsening indicates that the dendrite is still growing. When the dendrite continues to grow, the secondary dendrite grows continuously on the basis of the primary dendrite, and the secondary dendrite becomes more developed. However, the secondary dendrite is finer and weaker than the primary dendrite, similar to the trunk and the branches. Compare the trunk with the primary dendrite, and compare the branches with the secondary or tertiary dendrite. The trunk continues to grow, the branches are more developed, and the strength of the branches is weaker than the trunk. Therefore, the plastic deformation of secondary dendrites during laser pool solidification is the main cause for the accumulation change of dendrite orientation. High Fe content (15 wt.% Fe) could decrease the ratio of transverse temperature gradient to longitudinal temperature gradient, restrain the transverse growth of secondary dendrites, and cause dendrite orientation to be <001>.

The direct reason for the variation in the grain boundary angle is the change in the orientation of the crystal. The laser molten pool is unstable solidification, and any fluctuation in the solidification process leads to the change in orientation and the instability of the solidification interface, which leads to the formation of a low-angle grain boundary. Much research has been performed on the mechanism of low-angle grain boundary formation at home and abroad. Napolitano and Schaeferl [27] proposed the convergence–fault model for the formation of small-angle grain boundaries. Based on this model, because of the different solidification conditions of dendrite growth in different directions, the grain orientation of the dendrite changes accordingly, and a low-angle grain boundary is formed at the convergence location of the dendrites.

**Figure 6:**
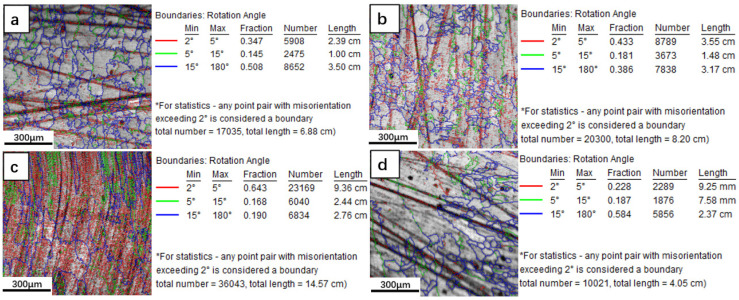
Grain boundary angle of Inconel 625 alloy cladding with different Fe content: (**a**) Inconel 625; (**b**) 5 wt.% Fe; (**c**) 10 wt.% Fe; (**d**) 15 wt.% Fe.

As shown in Figure 6, with the increase in Fe content, the proportion of low-angle grain boundaries in the cladding layer increases first and then decreases, which is 50%, 61.4%, 81%, and 41.6%. The frequency of low-angle grain boundaries is consistent with the variation in grain orientation.

**Figure 7:**
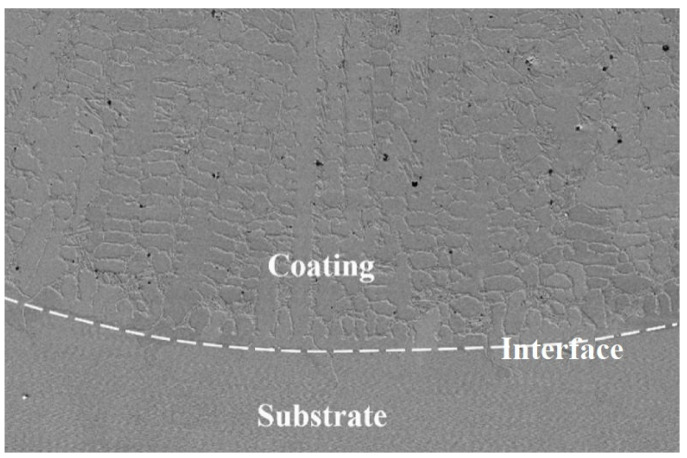
Solid–liquid interface between laser molten pool and matrix.

As illustrated in Figure 7, a transverse temperature gradient is formed at this point when the solid–liquid interface between the laser molten pool and the matrix is concave. The secondary dendrite grows laterally when the ratio of the transverse temperature gradient to the longitudinal temperature gradient is greater than a certain value [28]. The increase in Fe content may increase the ratio of transverse temperature gradient to longitudinal temperature gradient, which might disturb the growth of secondary dendrite and lead to transverse growth. The dendrites growing laterally converge with those dendrites growing along the <001> direction [29,30], increasing the proportion of low-angle grain boundaries. Moreover, the lower strength of the secondary dendrite leads easier deflection under the action of external forces (such as Marangoni convection, agitation, steam pressure, etc.), which causes the primary dendrite to lose its orientation and form a low-angle grain boundary [27], thereby increasing its proportion in the cladding layer. The grain boundary ratio for 15 wt.% Fe is reduced, which is due to the change in the thermodynamics and dynamics behavior during the solidification process of the laser molten pool, and the crystal orientation tends to be <001>. Therefore, with the increase in Fe content, the proportion of low-angle grain boundaries in laser cladding Inconel 625 alloy is firstly increased and then decreased.

### 3.3. Surface Hardness and Wear-Resistance Analysis

**Figure 8:**
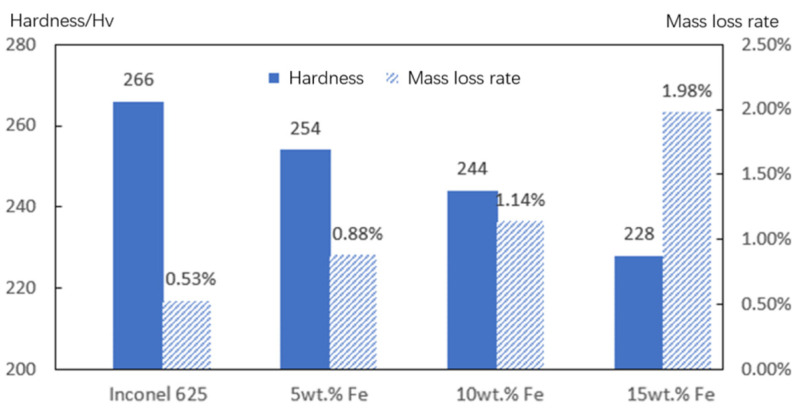
Hardness of the coating and the mass loss of friction wear.

An HXD-1000TMC/LCD Vickers microhardness tester was used to measure the hardness of the cladding layer with different Fe contents. The diamond indenter was loaded with 1.96 N and retained for 15 s. Every sample was randomly tested at 30 points in the area more than 1 mm from the edge, and the average hardness was regarded as the surface hardness of the cladding layer. The results indicate that there is a negative correlation between the surface hardness and the Fe content, as shown in Figure 8. On the one hand, with the increase in Fe content, the grains coarsen, and the fine grain enhancement is weakened at the maximal undercooling level of laser cladding. On the other hand, Inconel 625 is a solid-solution reinforced alloy of Nb, Mo, and other elements. The atomic radii of Fe, Nb, are Mo is shown in Table 3. The smaller Fe atoms can be substituted for Nb, Mo, etc., which can decrease the distortion of the lattice and weaken the strengthening effect of the solid solution. Therefore, the low dilution rate controlled by laser cladding Inconel 625 alloy is the main reason why the cladding layer has a high hardness.

An Ht-1000 high-temperature friction and wear testing machine was used for a 500 °C friction and wear test. The grinding ball was made of GCr15, the friction and wear-round sample had a diameter of 30 mm and thickness of 11mm. After polishing the surface of each cladding layer, the weight of the friction and wear samples was balanced to be equal by grinding the substrate, and the weight-loss ratio was compared. The friction and wear testing machine has 100 r/min, the friction radius was 10 mm, the load was 100 N, and the test time was 60 min. As shown in Figure 8, with the increase in Fe content, the wear resistance of the cladding layer was reduced, and the wear resistance was consistent with the hardness variation in the cladding layer. The higher the hardness of the cladding layer, the better the wear resistance. Controlling the low dilution rate of Fe can improve the wear resistance of the cladding layer at high temperature.

### 3.4. Corrosion Resistance Analysis

The cladding layer with 5 mm thickness was prepared by the laser multilayer cladding technique. To avoid the diffusion of matrix elements into the cladding layer and affecting the experimental results, 20 mm × 20 mm × 3 mm samples were taken from the outer surface of the cladding layer. After polishing, an electroheated molten salt furnace was used to conduct the molten salt corrosion experiment for 360 h at 200, 300, 400, and 500 °C. The composition of the molten salt is shown in Table 4. Five samples were taken from every cladding layer. After the corrosion experiment, the samples were washed with acetone in an ultrasonic cleaning machine, and the average weight loss was considered as the corrosion loss weight of the cladding layer.

**Figure 9:**
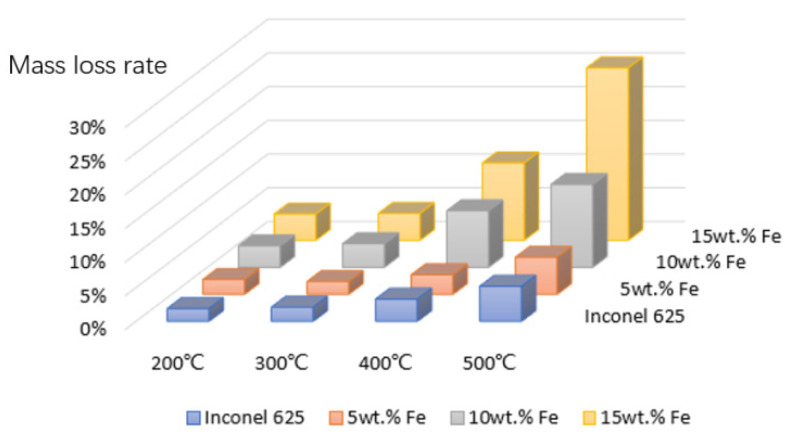
Weight-loss rate of Fe content in cladding layers at different temperatures.

Weight-loss rate of Fe content in the cladding layers at different temperatures is shown in Figure 9; with the increase in temperature and Fe content, corrosion resistance of the cladding layer was reduced; especially, when the Fe content was more than 5 wt.% of the cladding layer, the weight loss was significantly increased regardless of temperature. The weight-loss rate of Inconel 625 and 5 wt.% Fe cladding layers at different temperatures was small and nearly the same, and the corrosion resistance was good. This result indicates that the Fe dilution rate of laser cladding Inconel 625 alloy should be strictly controlled at less than 5 wt.% in order to guarantee the excellent anticorrosion performance of the cladding layer.

## 4. Conclusions

The influence of Fe content on the microstructure and performance of Inconel 625 alloy was investigated by adding different Fe contents into Inconel 625 alloy. The results show that it is very important to control the microstructure and performance of the cladding layer by controlling the Fe dilution rate in the laser cladding Inconel 625 alloy layer. The conclusions are as follows:

1. The preferred grain orientation <001> of the Inconel 625 laser cladding layer is first weakened, and then strengthened with the increase in Fe content. There is a positive correlation between the degree of grain orientation and the proportion of low-angle grain boundaries. The stronger the preferred grain orientation, the smaller the low-angle grain boundaries.
2. With the increase in Fe content, the phase of the Inconel 625 alloy cladding layer was not changed, but the solid solution of Nb, Mo, and other elements in the Inconel 625 alloy were decreased, the grains was coarsened, the fine grains was decreased, the surface hardness of the coating was decreased, and the wear resistance was decreased.
3. When Fe content was below 5 wt.%, the hardness, wear resistance, high temperature corrosion resistance, and the performance were similar. However, when Fe content exceeded 5 wt.%, the wear resistance and the corrosion resistance at high temperature were significantly reduced. Therefore, 5 wt.% Fe can be used as the reference limit for controlling the dilution rate for the laser cladding Inconel 625 alloy layer.

## Figures and Tables

**Table 1:** The composition of the base materials.

**Composition**	Fe	C	Mn	P	Si	Cr	Cu	Ni
**wt.%**	Bal.	0.200	0.410	0.014	0.210	0.060	0.165	0.050

**Table 2:** The composition of Inconel 625 alloy powders.

**Composition**	Ni	C	Mn	Mo	Si	Nb	Ti	Cr	Co	Fe
**wt.%**	Bal.	0.035	0.15	9.56	0.3	3.8	0.15	22	0.03	1.5

**Table 3:** Atomic radius.

**Atomic Species**	Fe	Nb	Mo
**Radius/Å**	1.24	1.43	1.36

**Table 4:** The composition of the molten salt.

**Composition**	NaCl	K_2_SO_4_	KNO_3_	NaNO_3_
**wt.%**	45	20	15	20
